# Recruitment, Retention, and Future Direction for a Heart Health Education and Risk Reduction Intervention Led by Community Health Workers in an African American Majority City

**DOI:** 10.1007/s40615-022-01329-z

**Published:** 2022-05-31

**Authors:** Julie Gleason-Comstock, Cindy Bolden Calhoun, Ghadir Mozeb, Cardell Louis, Alex Hill, Barbara J. Locke, Victor Harrell, Sadia Yasmin, Liying Zhang, John M. Flack, Nancy T. Artinian, Jinping Xu

**Affiliations:** 1 Department of Family Medicine & Public Health Sciences, 3939 Woodward Avenue, Detroit, MI USA; 2 Wayne State University School of Medicine, Detroit, MI 48201 USA; 3 Community Health Awareness Group, Inc., Detroit, MI USA; 4 Department of Anthropology, Wayne State University, Detroit, MI USA; 5 Department of Internal Medicine, Southern Illinois School of Medicine, Springfield, IL USA; 6 College of Nursing, Michigan State University, East Lansing, MI USA

**Keywords:** African American, Heart health education, Community health workers, Risk reduction, Blood pressure, Community Advisory Group, Urban health

## Abstract

Heart disease is a leading cause of death for African Americans. A community-academic partnership cross-trained community health workers to engage African American adults in a 6-month heart health education and risk reduction intervention. We conducted a one-group feasibility study using a one group (pre-posttest) design. A total of 100 adults were recruited from 27 zip codes in an African American majority city through community-based organizations (46%), churches (36%), and home visits (12%). Ninety-six percent were African American; 55% were female, 39% were male, and 6% were transgender. Their mean age was 44.6 years (*SD* = 15.9). Ninety-two percent had health insurance. Seventy-six percent of participants averaged blood pressure (BP) readings > 130/80 mmHg. Eleven percent of participants had a 30% or higher probability of developing cardiovascular disease in the next 10 years. Six-month follow-up was completed with 96% of participants. There were statistically significant increases in knowledge and in perception of personal risk for heart disease. However, slightly more participants (*n* = 77, 80.2%) had *BP* > 130/80 mmHg. The Community Advisory Group recommended expanding the intervention to 12 months and incorporating telehealth with home BP monitoring. Limited intervention duration did not meet longer term objectives such as better control of high BP and sharing risk reduction planning with primary care providers.

## Introduction

Heart disease is a leading cause of death in the United States and the State of Michigan [1]. In Detroit, an African American majority city and the largest city in Michigan, heart disease accounted for 31.6% of all deaths in 2013 [2]. High blood pressure (BP) is a primary risk factor for heart disease in African Americans [3] as well as having a higher prevalence among African American adults (57.1%) than non-Hispanic white (50.2%) and Hispanic adults (50.1%) [4].

Heart disease is considered largely a preventable disease because most risk factors are modifiable through lifestyle changes (reduced smoking, weight loss, increased physical activity) and bringing BP under control. Wayne State University (WSU) School of Medicine (SOM) Department of Family Medicine & Public Health Sciences (DFMPHS) Investigators had experience and published two prior studies [5, 6] addressing BP control with the Detroit African American community. DFMPHS and the Cardiovascular Research Institute (CVRI) formed a community-academic partnership with Community Health Awareness Group, Inc. (CHAG) to address heart health for Detroit residents. CHAG is a non-profit, minority-operated, community-based organization serving African American and Greater Detroit residents with health risks. WSU evaluators and researchers and CHAG have worked together for 25 years in a community-academic partnership, addressing health disparities in HIV and Hepatitis C, and expanded that research activity to cardiovascular disease and heart health.

The CHAG Community Advisory Group (CAG) is comprised of about a dozen Detroit residents, primarily African-American, about half male and half female, with LGBTQ + representation, and has met quarterly for over a decade to advise CHAG on prevention and intervention programs, which were expanded to include heart health. Background information on the importance of BP measurement in heart health was provided through explanation of results and lessons learned from Investigators’ prior study with the City of Detroit adult primary care clinic [5]. The CAG suggested an abbreviated name, “Heart*B*,” as the theme for explaining the new outreach project to Detroit residents. The purpose of this paper is to describe foundations of the heart health education pilot project, participant recruitment and retention, and proposed future direction.

Wayne State University Institutional Review Board (IRB) Behavioral (B3) (IRB #023015B3F) approval for behavioral, educational, and social science research was received in April, 2015. Participant recruitment began after the IRB approval.

## Community Health Workers and Training

Use of community health workers (CHW) is an important public health strategy to address health equity. Academic-community partnerships with trained CHW, focusing on risk reduction, have been effective for short-term health outcomes. All the Heart*B* CHW were African American, and they served as trusted ambassadors for the project [7]. The CHW had 6 to 23 years of Detroit community outreach experience and all had completed Michigan Department of Health & Human Services (MDHHS) HIV Test Counselor Certification.

In preparation for the intervention, CHW and project staff completed two web-based and one on-site training. The online training from the Centers for Disease Control and Prevention (CDC) 2014 *A Community Health Worker Training Resource for Preventing Heart Disease and Stroke* (https://cdc.gov/dhdsp/programs/spha/chw_training/pdfs/chw_training.pdf) for which CHW received CEU. Each of the 15 chapters of the CDC training resource contained heart health content, learning objectives, information, and activities to increase skills in prevention of heart disease and stroke, pre- and post-tests, and community presentation and resource materials. The presentations were previewed for cultural appropriateness at evaluation and Community Advisory Group meetings, and recommended for implementation prior to their use with project participants.

**Fig. 1:**
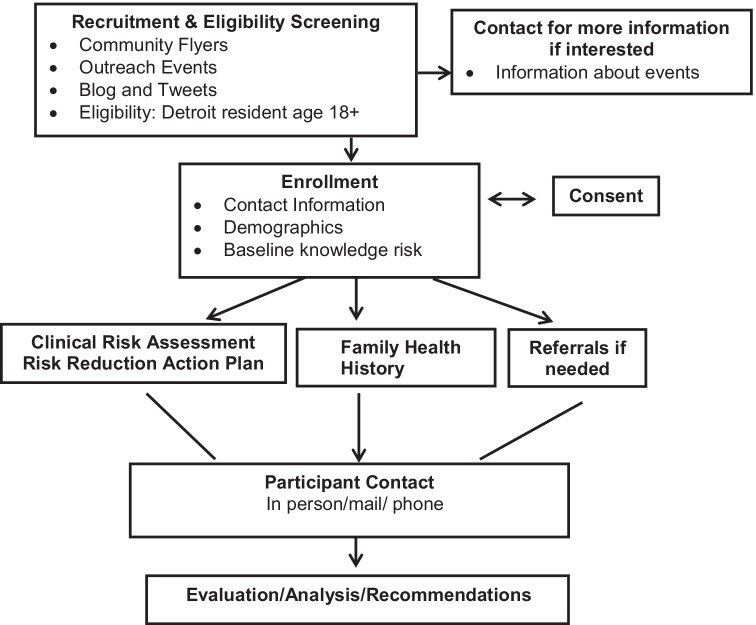
Project protocol

A second training was on-site and focused on protocol and evaluation activities described in the “Methods” section in Fig. 1. This interdisciplinary training was three half-days and conducted at CHAG.

The third training was a self-paced, using *With Every Heartbeat is Life: A Community Health Worker’s Manual on Heart Disease for African-Americans* (*WEHL*) (https://www.nhlbi.nih.gov.files/docs/resources/heart/aa_manual.pdf) created especially for African American communities by the National Heart, Lung and Blood Institute (NHLBI). *WEHL* was first produced in 2008 and was updated as a new NHLBI publication in 2020. The *WEHL* Community Education Strategy of providing education and screening was utilized by Heart*B* and incorporated in Table 1. *WEHL* community education goals for the African American community were (a) increasing knowledge about heart health, (b) increasing positive attitudes to make lifestyle changes, (c) increasing adoption of healthy lifestyle behaviors, (d) tracking participants’ clinical data, and (e) referring participants with elevated levels to health care professionals to confirm if levels are high. Informed consent was imbedded in the Heart*B*—*WEHL* strategy to assure confidentiality and fidelity of activity. Each of the four CHW created a group presentation using the CDC and NHLBI materials to teach a content area (nutrition, exercise, smoking, or blood pressure control) for participant engagement.

**Table 1:** Detroit Heart*B* community engagement logical model

Inputs	Activities	Outputs	Participant outcomes		
			Short-term	Intermediate	Long-term
City of Detroit residents, community organizations, churches Community Health Awareness Group, Inc. (CHAG): Community Advisory Board (CAG), community health workers (CHW), Nurse Community-Academic Partnership: CHAG & WSU Depts Family Medicine & Public Health Sciences; College of Nursing Resources (Manuals): CDC (2014) CHW *Training for Preventing Heart Disease;* NHLBI (2008) *With Every Heartbeat is Life: for African Americans* Funding: Detroit Medical Center Foundation, WSU Cardiovascular Research Institute	CAG meetings CHW orientation and protocol training WSU IRB Social Behavioral Training (CITI) Health literacy screening Cardiovascular risk assessment Blood pressure (BP) monitoring Risk reduction education/plans Family health history portrait Health referral as needed	# CHW trained and conducting outreach #Participants consenting #Participants completing baseline and 6-month follow-up assessments % Enrolled at medium to high risk for heart disease %Partici-pating in BP monitoring # Sharing health history with family/significant others and providers	Increased knowledge heart health and disease risk Increased perception of own risk for CVD Increased understanding family history effects on personal health Increased personal monitoring high BP Participant perceived intervention importance and satisfaction	Increased client awareness of risk behaviors Increased sharing risk reduction plan with primary care provider Decreased BP readings Increased BP monitoring Increased linkage to primary care provider	Increased healthy behaviors (dietary, exercise, reduced smoking) Increased controlled high BP Decreased heart disease for Detroit African Americans

Assumptions/contextual factors:

Funding will be secured throughout the course of the project

CHW staff with outreach skills will facilitate linkage to primary care

Risk reduction planning will be shared with primary care providers

### Detroit HeartB Objectives

The primary objective of Heart*B* was to reduce heart disease risk in Detroit by providing a heart health education intervention to increase participants’ knowledge of heart disease prevention and work to facilitate expansion of risk reduction skills. For this 6-month intervention, expected short-term program outcomes presented in the Logic Model were assessed at enrollment (baseline) and follow-up (6 months) visits. The short-term outcomes focused on increasing participant knowledge and skills around heart disease and high BP prevention, and their perception of personal risk for cardiovascular disease, as well as better understanding of family history effects on personal health.

Community health workers were introduced to the online Health & Human Services “Family Health Portrait” (https://familyhistory.hhs.gov/FHH/html/?action=create which was piloted during training sessions. CHW expressed concern about the complexity (online access, file retrieval, multiple variables), so evaluators also provided a paper version of American Heart Association’s Family Health History Tree (https:// www.IKnowDiabetes.org), which CHW found more useful.

CHAG staff were asked to evaluate their training experience for the project. Seven evaluations were completed (four CHW, the nurse, and two administrative staff who also conducted outreach). The staff perceived structured on-site training, ongoing mentoring, and observational feedback as important. Their favorite training was the behavioral and clinical protocols; their least favorite was the WSU Institutional Review Board (IRB) Collaborative Institutional (CITI) Social/Behavioral training on consent, evaluation, and research (Streater, 2015).

## Methods

### Recruitment and Protocol

Participants were recruited through flyers, outreach contact, and other CHAG program services. In fall of 2015, CHAG held a kick-off event at their central office. Printed flyers were distributed by the CHW to community organizations and churches throughout Detroit. The CHAG project team, which included the Executive Director and Prevention Director, community project nurse, and CHW, welcomed potential Detroit participants. Interested persons were consented and completed the enrollment process at that event. The event also provided the project team with a pilot session for data collection and intervention procedures. All project recruitment was completed in spring, 2017.

During their first session (enrollment), participants were introduced to the project, gave informed consent, and met with the CHW to complete the baseline assessments (heart health knowledge, perceived risk for developing heart disease, health literacy, and family health history). After introducing the importance of family medical health and its impact on health, participants were introduced to family history tree options for them to complete and share with their family and their health care providers if they wished.

Following collection of family history information, participants met with the project nurse for clinical assessments (weight, height blood pressure, and medical history) and to discuss their cardiovascular risk factors. The nurse had a separate clinic room. Before BP measurement, each individual was asked if they had smoked, had a cup of coffee or climbed a flight of stairs in the last 30 minutes; if so, they had to wait 30 minutes before the nurse could proceed to taking their BP. Before taking BP, the individual had to be wearing non-restrictive clothing, have rested for five minutes, have their feet on the floor, their back supported and their arm supported at heart level. The nurse determined the right BP cuff size to ensure accurate measurement. In the clinical assessment for blood pressure, the nurse took at least two blood pressure readings at least 10 s apart. If they differed by 5 mmHg or more (either systolic or diastolic numbers), she continued taking blood pressure readings until there were two readings that only differed by 5 mmHg or less. The last two readings were recorded in the participant chart. The two obtained BP readings were averaged.

Based on the clinical assessments and medical history, the Framingham Heart Study Cardiovascular Risk Assessment primary care tool [8] was used to provide the participants with their heart age and CVD risk for developing heart disease in the next 10 years. The intervention was for ages 18 years and older, so CVD risk assessment was included for participants age 18 to 81 years in order to reflect the study population.

The project nurse and CHW reviewed their key risk factors with the participant and guided them in developing an action plan to lower their risk. All participants received a copy of the risk factors, blood pressure reading, heart age, CVD risk scores, and recommended actions.

The community-academic project team conducted an implementation project flow assessment during the first community enrollment event at CHAG. Project procedures were evaluated including length of time for participants to complete each of the three major “project stations,” i.e., consent and enrollment, knowledge about heart disease and family history, and CVD risk assessment and creation of an action plan. To accommodate multiple participants at a given location, the project was designed to be completed as a series of separate stations. Following the first station, participants moved to other stations in a flexible rotating order. The average amount of time for each participant to complete assessment forms with community health workers and the nurse at each of the stations was close to an hour. Taking into account additional considerations such as workflow, the three-page consent, participant contact forms, and risk reduction discussion, project staff estimated it took over an hour to enroll each person, described as follows.

- The enrollment process, which included information about demographics and access to medical care, took an average of 4.41 min, or about 5 min.
- Baseline assessments, which included collecting data about knowledge, perceived risk, health literacy, and family health history, took an average of 15.16 min, or close to 20 min.
- CVD Clinical Risk Assessment and creation of a risk reduction action plan took an average of 17.49 min, close to 20 min.

CHW completed project staff logs for each subsequent participant encounter (i.e., individual or group in-person, mail and phone) which were incorporated into individual participant files. The program protocol is shown in Fig. 1.

Immediately following the pilot kick-off event and project flow assessment, the evaluators facilitated a group discussion with program staff to improve data collection and program delivery efficiency. Based on this discussion, program staff reworked the program enrollment sequencing to increase participant flow through project activities and decrease participant waiting time. This included establishing multiple protocol stations, i.e., after completing consent, participants could move to the enrollment station, participants could move directly to the nurse station for clinical risk assessment, or to another session with CHW regarding family health.

### Measurement Tools Used for Data Collection

Participant health literacy was assessed through reading a nutritional label [9]. Participants answered six questions about a nutrition label for a serving of ice cream. Zero to 1 correct responses indicated limited literacy, 2–3 correct indicated possible limited literacy and 4 -6 indicated adequate literacy.

The CVD Clinical Risk Assessment tool [8] was used to determine participant risk for a cardio-vascular event in the next 10 years. Probability for a CVD event was determined using a set of non-lab dependent, sex-specific risk factors based on age, systolic blood pressure, diabetes diagnosis, Body Mass Index (BMI) (height and weight), and smoking status. The tool provided a numeric score which determined both the likelihood of a CVD event as well as a comparable heart age.

Two methods were used to assess knowledge. The first method was a self-reported rating of how much they thought they knew about high blood pressure and heart disease: not very much, a little, some, and a great deal. The second method was assessed through 20 multiple choice and true/false questions from the Hypertension Evaluation of Lifestyle and Management (HELM) knowledge scale [10] and the Heart Disease Fact Questionnaire [11]. Both item formats (i.e., multiple choice and true/false) were used to compensate for those who do better with one test format than another.

Perceived risk was defined using a 5-point scale in which participants rated how likely they thought their chances are of developing heart disease in the next 10 years were. Understanding the relationship between family history and personal health was assessed using a 5-point rating scale, with higher ratings reflecting greater perceived risk of developing in the next 10 years [12]. Four health conditions were rated as follows: high blood pressure (HBP), diabetes, high cholesterol, and heart disease.

### Statistical Analysis

Data were summarized using counts and percentages for categorical variables and means and standard deviations for continuous variables. Frequency distributions for variables of interest were calculated for both pre-survey and post-survey. Descriptive analysis for sociodemographic characteristics, risk behaviors and health conditions, and outcomes between baseline and 6-month follow-up was conducted. The differences between pre- and post-survey were examined using related samples nonparametric tests including Wilcoxon signed-rank test for continuous outcomes (e.g., knowledge) and the McNemar test for categorical outcomes (e.g., smoking). All statistical analysis was performed using IBM SPSS Statistics 27 (https://www.ibm.com).

## Results

### Participant Baseline Characteristics

**Fig. 2:**
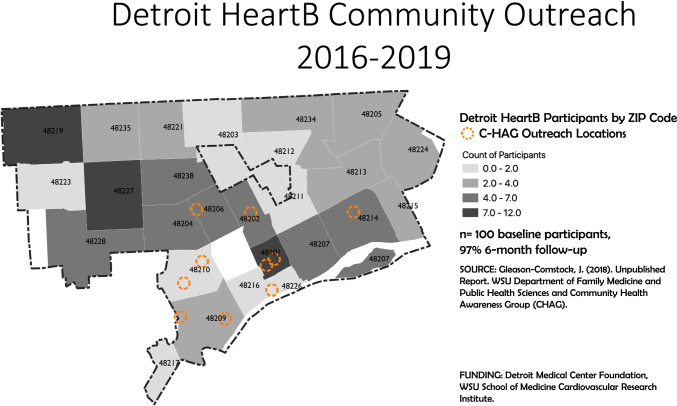
Detroit Heart*B* community outreach 2016–2019

One hundred residents from 27 zip codes throughout the City of Detroit were enrolled into the program. The CHAG outreach included community-based organizations (46% of participants), churches (36%), and home/outreach van visits (12%). Please see Fig. 2.

Among the study participants, 96% were African-American, 55% were female, 39% were male, and 6% were transgender (male to female). Almost two-thirds (63%) of participants reported an annual household income of under $25,000. The majority (78%) were not currently married. About two-thirds (68%) of participants were scored as having adequate health literacy, 17% with possible limited literacy, and 15% with a high likelihood of limited literacy. See Table 2 for selected detail of baseline demographics by gender.

**Table 2:** Detroit Heart*B* baseline demographics

Characteristic	Female *N* = 55	Male *N* = 39	Transgender *N* = 6	Total *N* = 100
Race/ethnicity				(100%)
African American/Black	53	37	5	95
Other	2*	1**	0	3
African American/Hispanic	0	0	1	1
White	0	1	0	1
Age mean (*SD*), range	46.8 (*SD* = 16.1) 18–81 years	44.0 (*SD* = 15.53)19–80 yrs	28.7 (*SD* = 4.08), 26–36 yrs	44.6 (*SD* = 15.9) 18–81 yrs
Education *n* (%)				
Less than high school	11(20%)	6 (15.4%)	1 (16.7%)	18 (18.0%)
High school graduate (GED)	21(38.2%)	18 (46.2%)	4 (66.75%)	43 (43.0%)
Trade school/college	23 (41.8%)	15 (38.5%)	1 (16.7%)	39 (39.0%)
Employment *n* (%)				
Working	23 (41.8%)	21 (53.8%)	1 (16.7%)	45 (45.0%)
Not working	32 (58.2%)	17 (43.6%)	5 (83.3%)	54 (54.0%)
Missing/no responses	0 (0.00%)	1(2.6%)	0 (0.00%)	1 (1.0%)
Health care insurance *n* (%)				
Yes	52 (94.5%)	37 (94.9%)	6 (100.0%)	95 (95.0%)
No	2 (3.6%)	2 (5.1%)	0 (0.00%)	4 (4.0%)
Missing/no responses	1(1.8%)	0(0.00%)	0 (0.00%)	1 (1.0%)
Primary care provider *n* (%)				
Yes	48(88.9%)	38 (97.4%)	5 (83.3%)	91 (91.0%)
No	6 (11.1%)	1(2.6%)	1 (16.7%)	8 (8.0%)
Missing/no responses	1 (1.9%)	0 (0.00%)	0 (0.00%)	1 (1.0%)

*Mixed race **American Indian, unknown

### Cardiovascular Disease Risk Factors

The majority of participants (81.8%, *n* = 81) reported they did not have a father/brother who had a heart attack before the age of 55. About 15% (15.2%, *n* = 15) said yes, and 3% (*n* = 3) did not know. Similarly, in regard to having a mother/sister who had a heart attack before the age of 65, the majority (81.8%, *n* = 81) said no, 14.1% (*n* = 14) said yes, and 4% (*n* = 4) did not know. There were no significant differences between male, female, and transgender participants.

According to the Cardiovascular Disease Risk algorithm, [8] about half of participants (54%) had less than a 10% probability of a cardiovascular event in the next 10 years. About a quarter of participants (26%) had between a 10 and 19% probability of a CVD event in the next 10 years. However, a fifth had a 20% or greater probability of a CVD event in the next 10 years. Please see Table 3.

**Table 3:** Probability of CVD event in the next 10 years

Percent risk	Frequency (percent)
Below 1%	4 ( 4.0%)
< 10%	50 (50.0%)
10–14%	11 (11.0%)
15–19%	15 (15.0%)
20–29%	9 ( 9.0%)
30% or higher	11 (11.0%)

### Participant 6-Month Follow-up

Ninety-six participants (96%, *n* = 96) participants completed the 6-month follow-up.

### Heart Health Knowledge

For the 96 participants who completed the 6-month follow-up, there was a significant increase in heart health knowledge, from 12.0 (*SD* = 2.35) correct items at baseline to 13.1 (*SD* = 3.66) at 6 months (*p* < 0.001*)*.

### Perception of Cardiovascular Risk

Participants were asked to respond, on a scale of 1 = not very likely to 5 = very likely, “how likely do you think your chance of developing each of the following health conditions is in the next 10 years: (a) high blood pressure, (b) high blood sugar (diabetes), (c) high cholesterol, or (d) heart disease, heart attack, or stroke.” There was a statistically significant decrease in perception of personal risk for heart disease, heart attack, or stroke, from 1.86 (*SD* = 1.13) at baseline to 1.72 (*SD* = 1.10) at 6 months (*p* < 0.05).

### Behavioral Risk Factors

Although BMI decreased slightly from 31.3 (*SD* = 9.8) at baseline to 30.3 (*SD* = 10.5) at 6 months, it was not statistically significant. There were no statistically significant changes between baseline and the 6-month follow-up in self-reported behaviors for exercise, nutrition, or smoking cessation.

### Systolic and Diastolic Blood Pressure

There was a small but non-significant change in systolic blood pressure (SBP), i.e., 130.87 mmHg (*SD* = 21.84) at baseline to vs. 130.82 mmHg (*SD* = 18 0.89) at 6-month follow-up (*p* = 0.972). Similar findings were noted for diastolic BP, i.e., 83.93 mmHg (*SD* = 13.08) at baseline vs. 82.19 mmHg (*SD* = 9.19) at 6-month follow-up (*p* = 076.). At 6-month follow-up, slightly more participants (*n* = 77, 80.2%) had BP > 130/80 mmHg.

### Participant Rating of Importance of HeartB Activities

Participants were asked to score their perception of importance of ten Heart*B* intervention activities in which they had participated. As shown in Table 4, on a scale of (1) very important to (5) not important, “Creating a plan to reduce my risk for heart disease” scored the highest (*M* = 1.93, *SD* = 0.86), followed by “Learning my heart age” (*M* = 1.91, *SD* = 1.06) and “Getting my blood pressure checked” (*M* = 1.48, *SD* = 0.63).

**Table 4:** Participant scoring of importance with Heart*B* activities

Item	Mean
Creating a plan to reduce my risk for getting heart disease	1.93 (*SD* = 0.86)
Learning my heart age	1.91 (*SD* = 1.06)
Getting my blood pressure checked	1.48 (*SD* = 0.63)
Learning about heart disease and high blood pressure	1.35 (*SD* = 0.54)
Completing my family health history tree	2.74 (*SD* = 4.52)
Talking with project staff about what I can do to reduce my risk for heart disease	2.03 (*SD* = 0.97)
Learning how to read nutrition label	3.34 (*SD* = 1.54)
Being able to stop by between appointments to talk to the nurse of other program staff	3.24 (*SD* = 1.44)
Getting phone calls from project staff between program visits	3.05 (*SD* = 1.19)
Getting a referral to a primary care doctor/clinic	3.56 (*SD* = 1.53)

## Discussion

### Limitations

The project relied on CHW who had extensive previous experience with urban community outreach and evaluation in the African American community, and many organizations do not have this expertise.

Short-term objectives of increasing participant knowledge and perception of personal heart health risk were met. However, the limited intervention duration did not provide the opportunity to meet longer term objectives such as better control of high BP and sharing of risk reduction planning with participants’ primary care providers.

### Future Direction

The Heart*B* intervention challenged CHAG to broaden from a primary focus on HIV/AIDS and Hepatitis C to include heart health education, prevention, and risk reduction. As an extension of the training, the CHW developed a set of heart healthy educational presentations based on the CDC and NHLBI training resources that were used to enhance program activities. The presentations covered blood pressure monitoring, nutrition, physical activity, and smoking cessation. The forms, tools, and procedures developed for this project are fully established and can be used future program expansion and recruitment.

The CAG suggested the 6-month intervention was insufficient to address participant risk factors to address chronic disease management. For future interventions, they recommended providing mobile home BP monitors with carrying cases, as participants may be in transit between sites during the time they need to monitor their BP. They suggested the use of telehealth in addition to home visits, and the option of completing online questionnaires for follow-up surveys. Potential CHW outreach and follow-up incorporating CHAG mobile clinic vans were also noted.

Evaluators followed up on CAG recommendations to offer participants the option of online forms and participation. Detroit Heart*B* consent, enrollment, and follow-up forms were uploaded into Qualtrics for use on smartphones or iPads for future projects. CHW presentations are now also available as PowerPoint presentations for future web-based applications.

The CAG continues to receive updates and make recommendations for expansion of Heart*B*. Meetings were suspended in 2020 due to COVID-19 but resumed in September of 2021. During the September 2021 and April 2022 meetings, both expansion of Heart*B* and the impact of the COVID-19 pandemic were discussed were discussed by CAG members and CHAG staff. National case surveillance of the COVID-19 epidemic from January to May of 2020 showed African-Americans disproportionately affected by COVID infection and deaths. In that same time period, in Michigan, where African-Americans are 14% of the State’s population, African-Americans were 33% of COVID-19 cases and 41% of deaths, with Southeast Michigan/Metropolitan, in which Detroit is located, as a hotspot (https://www.Michigan.gov/coronavirus). The CAG noted African American communities continued to be impacted by long-term COVID-19 as well as hypertension, and suggested interdisciplinary health teams which included CHW, as well as patient engagement in prevention, risk reduction, and treatment were key [13, 14].

The CAG also suggested there needed to be more discussion about how cardiovascular disease may intersect with COVID-19 and its long-term effects. Research has shown it is unclear whether uncontrolled BP is a risk factor for acquiring COVID-19, or whether uncontrolled BP among patients with hypertension is more or less of a risk factor [15]. These implications for chronic disease management suggest the need for ongoing expansion of community and academic research partnerships to address social determinants and heart health for African-American adults [16].
